# A Celiac Disease Phenotype After Checkpoint Inhibitor Exposure: An Example of Immune Dysregulation After Immunotherapy

**DOI:** 10.14309/crj.0000000000000158

**Published:** 2019-08-08

**Authors:** Joud Arnouk, Don Mathew, Ethan Nulton, Vikrant Rachakonda

**Affiliations:** 1 Department of Internal Medicine, UPMC McKeesport Hospital, McKeesport, PA; 2 Department of Gastroenterology, Hepatology and Nutrition, UPMC Presbyterian Hospital, Pittsburgh, PA

## Abstract

Celiac disease is characterized by duodenal inflammation after exposure to gluten. Checkpoint inhibitors are antibodies that inhibit the inhibitory signals of the cytotoxic T lymphocytes to enhance antitumor responses. A 79-year-old man with an unknown history of celiac disease underwent treatment with pembrolizumab for recurrent left maxillary melanoma. He subsequently developed diarrhea and weight loss. Serology was positive for anti-tissue transglutaminase immunoglobulin A. Upper endoscopy revealed duodenal villous atrophy, which was confirmed on biopsy. A gluten-free diet was not tolerated, and symptoms resolved with withdrawal of pembrolizumab and steroid administration for another medical reason.

## INTRODUCTION

Celiac disease (CD) is a malabsorptive immune-mediated disease characterized by local inflammation in the duodenum due to intolerance of a gluten-rich diet in patients with a genetic predisposition.^1^ Immune checkpoint inhibitors (ICPIs) have been widely used in the management of many cancers by blocking the inhibitory receptors on cytotoxic T cells, leading to immune stimulation.^2^ Although colitis and hepatitis are known gastrointestinal toxicities related to immunotherapy, one case has described the association between CD and ipilimumab.^2,3^ We present the first case of a new-onset CD after exposure to pembrolizumab, a programmed cell death protein 1 (PD-1) inhibitor.

## CASE REPORT

A 79-year-old man with a history of stage IIa left maxillary superficial melanoma developed local recurrence 2 years after initial resection. The patient had an unknown history of CD. He was treated with local radiation and 200 mg of intravenous pembrolizumab every 3 weeks. One week after initiating the therapy, he developed loss of appetite and episodic, twice daily, watery, and nonbloody diarrhea. His symptoms progressed after 4 months of therapy with abnormal laboratory findings of hemoglobin 10.4 g/dL, potassium 3.1 mEq/L, albumin 2.4 g/dL, vitamin D 25-OH 16 ng/mL, and zinc 54 mcg/dL.

**Figure 1.**
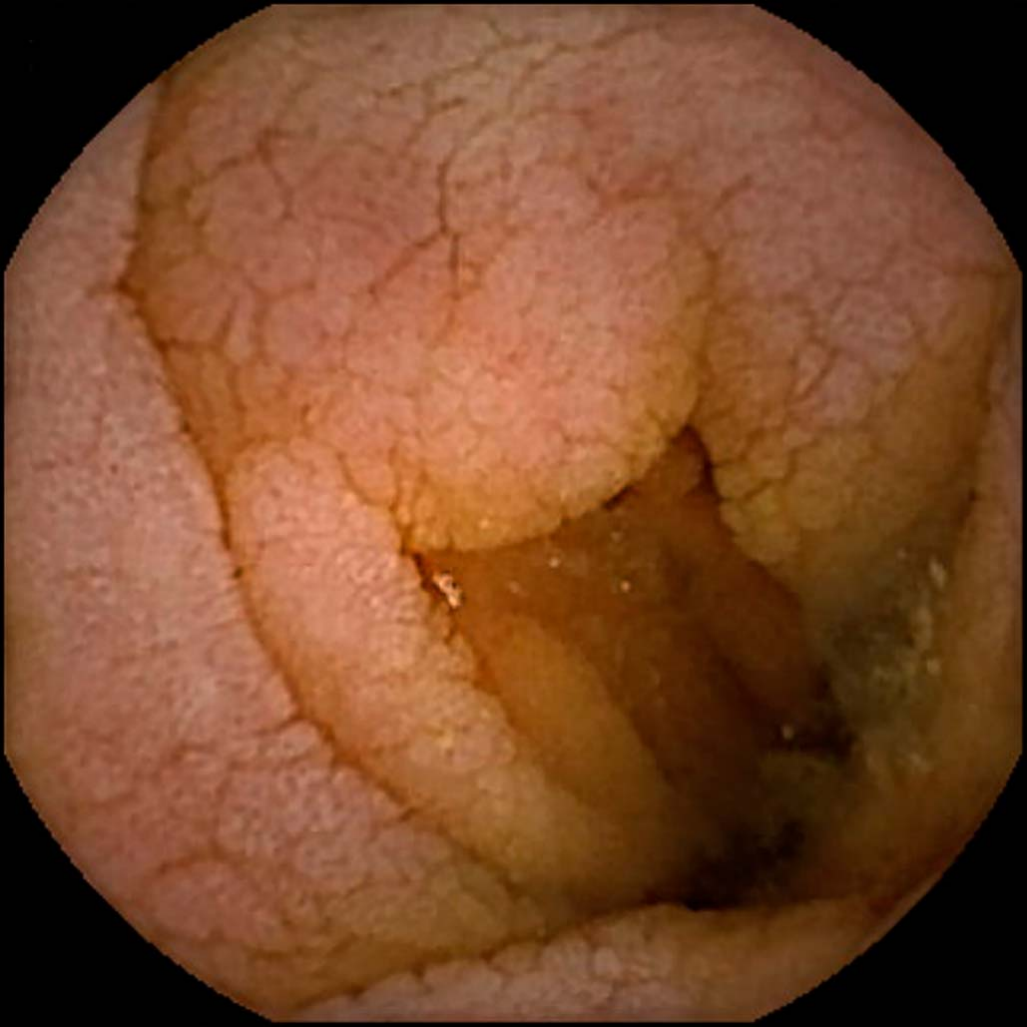
Capsule endoscopy image showing villous atrophy at the level of the second part of the duodenum.

**Figure 2.**
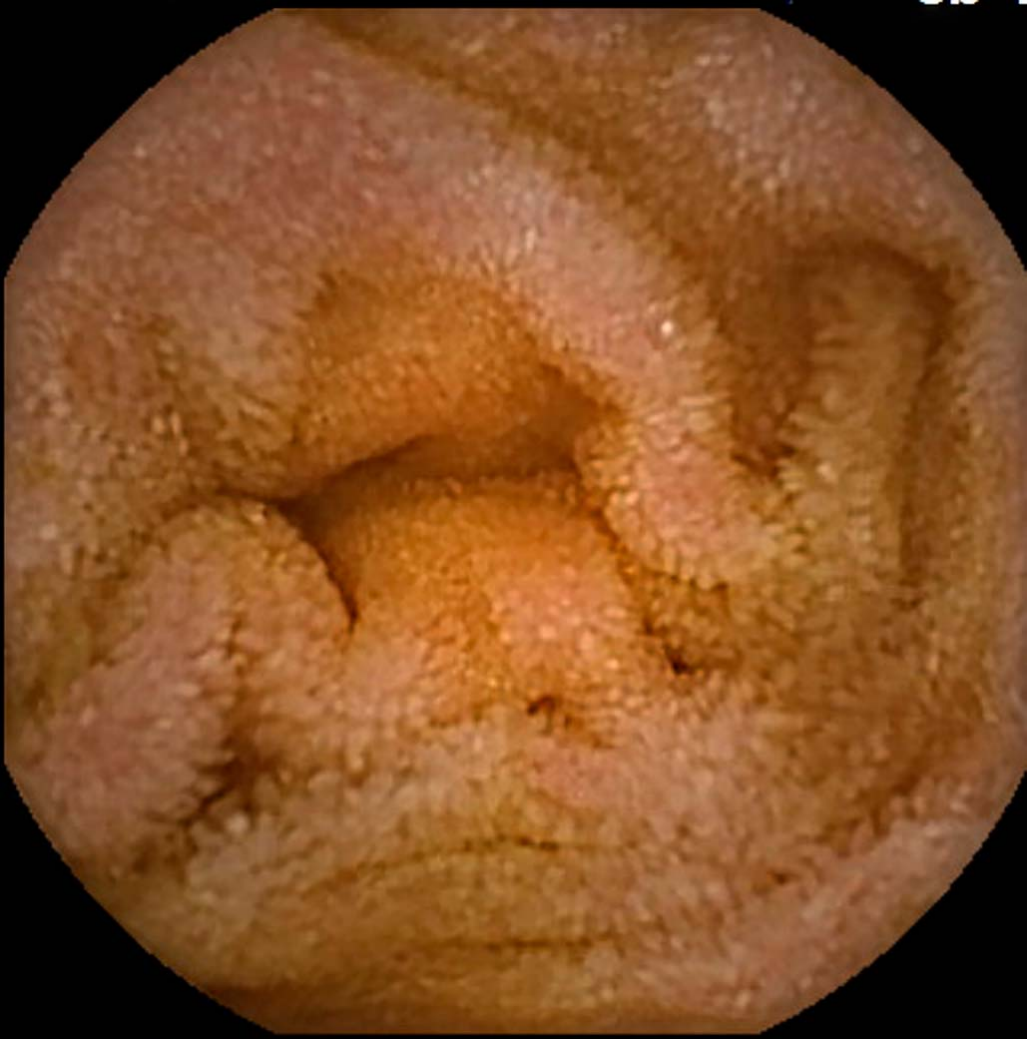
Capsule endoscopy image showing intact distal intestinal mucosa.

**Figure 3.**
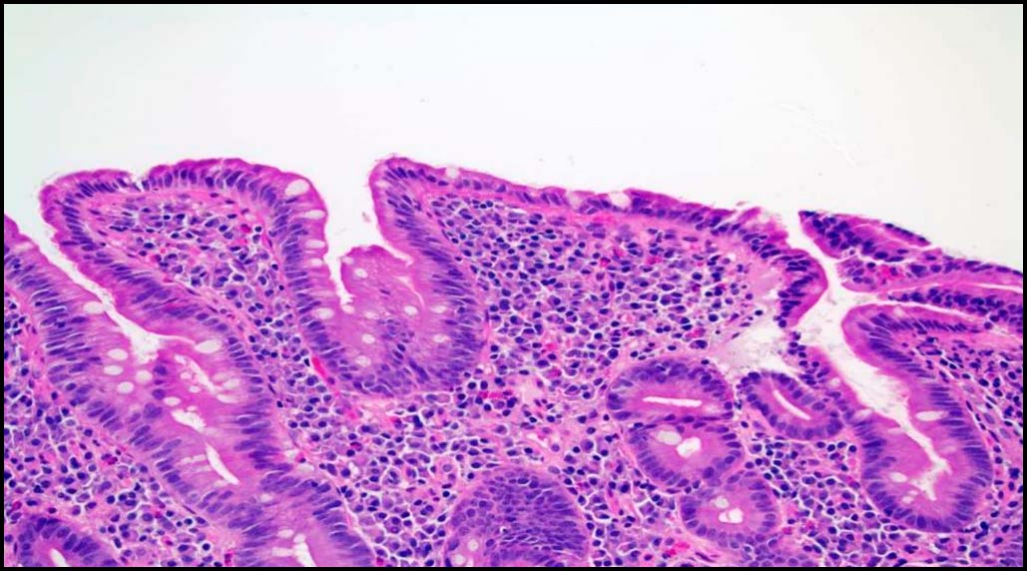
Duodenal biopsy showing lamina propria expansion, villous atrophy, and increased intraepithelial lymphocytes.

The patient was referred to a gastroenterologist. Stool studies including fecal leukocytes, lactoferrin, stool cultures, and *Clostridium difficile* were normal. The patient underwent a colonoscopy, which demonstrated normal-appearing mucosa with normal random biopsies. An upper endoscopy to the second part of the duodenum revealed a small antral erosion and normal-appearing duodenal mucosa. A wireless capsule endoscopy performed 1 week later to rule out enteritis revealed villous blunting in the second part of the duodenum. This was overlooked in the upper endoscopy (Figure 1). As opposed to the upper endoscopy, capsule endoscopy revealed pronounced villi in the ileum (Figure 2). Duodenal biopsy, however, revealed lamina propria expansion, villous atrophy, flat mucosa, and increased intraepithelial lymphocytes classified as type IIIc as per Marsh classification (Figure 3).

Serologies for CD revealed anti-tissue transglutaminase immunoglobulin A (IgA) of 59 U/mL, anti-gliadin IgA of 28 U/mL, and serum IgA of 213 mg/dL. A gluten-free diet was initiated; however, the patient was not able to afford it for more than 3 weeks because of the cost. Subsequently, his symptoms of weight loss and diarrhea worsened because of lack of adherence.

After multiple hospital admissions, pembrolizumab was discontinued 4 months after the initial dose. The patient was concurrently started on systemic steroids (hydrocortisone 10 mg in the morning and 5 mg at night) for postural hypotension. While on steroids, his symptoms of diarrhea resolved, gaining 20 pounds in 1 month after initiating therapy. The patient was lost to follow up because of multiple hospitalizations, and no repeat serologic or endoscopic studies were performed.

## DISCUSSION

CD is suggested by a combination of clinical characteristics and positive serology for celiac antibodies (tissue transglutaminase [TTG] antibody IgA is 95% sensitive and 95% specific). It is confirmed with villous blunting and mucosal inflammation on biopsy.^1^ CD has a wide variety of intraintestinal and extraintestinal manifestations; it can also be a silent disease in many patients.^1,4^ CD usually responds well to the elimination of gluten from the diet.^1^ However, patients may respond to systemic steroids when a gluten-free diet is not tolerated or in refractory cases.^1^

Diarrhea can be the main presenting symptom of the gastrointestinal tract toxicity due to immunotherapy.^5^ It can be a self-limiting, infectious, or immune-related adverse event.^5^ The American Society of Clinical Oncology has classified ICPI-related diarrhea into 4 grades. Grade 1 is defined as < 4 stools over the baseline in 24 hours. Grade 2 is defined as 4–6 bowel movements over the baseline in 24 hours. Grade 3 is defined as more than 7 stools over the baseline in 24 hours. Diarrhea associated with life-threatening consequences is grade 4.^2^ The recommended management of diarrhea in those taking immunotherapy is based on the same grading system with symptomatic management for grade 1. For grade 2, stool studies, diagnostic endoscopies, and treatment with steroids are indicated. The stool studies routinely obtained are fecal leukocytes, lactoferrin, calprotectin, stool cultures, and *C. difficile* testing. Pembrolizumab would also be held temporarily for grade 2. Grade 3 may require hospitalization, corticosteroids administration (initial dose of 1–2 mg/kg/d prednisone or equivalent), and infliximab for nonsteroidal cases along with temporarily hold of pembrolizumab. It can be resumed if diarrhea returns to grade 1. Permanent discontinuation of pembrolizumab and tapering steroids or infliximab are indicated for grade 4 diarrhea.^2^

PD-1 is a protein receptor expressed on T cells, and PD-1 ligands (PD-L1) are expressed on tumor cells.^6^ PD-1/PD-L1 blockade with pembrolizumab leads to interruption of the inhibitory signals and upregulate the stimulatory signals to reactivate the immune T cells.^6,7^ This activation may involve one or multiple organs, leading to serious adverse events known as immune-related toxicities.^5^ Multiple case reports have identified the association between ICPI toxicities and immune-related gastrointestinal diseases such as enteritis, esophagitis, hepatitis, and colitis.^2,5,8-10^ However, in this case, a new type of toxicity is reported, which will broaden the differential for any immunotherapy-related diarrhea in the future.

It is not clear whether pembrolizumab withdrawal or steroid administration or even both helped with the resolution of symptoms as both events happened concurrently. More studies are recommended to illustrate the exact mechanism.

The usage of ICPIs has quickly emerged and expanded to treat many cancers. Their complete safety and toxicity profile are still to be identified. The authors advise health care providers to take into consideration etiologies other than pembrolizumab-induced colitis or enteritis in the evaluation of diarrhea in the setting of new-onset immunotherapy.

## DISCLOSURES

Author contributions: J. Arnouk wrote the manuscript and is the article guarantor. D. Mathew, E. Nulton, and V. Rachakonda edited the manuscript.

Financial disclosure: None.

Informed consent was obtained for this case report.
